# Magnetic phases of spin-1 spin–orbit-coupled Bose gases

**DOI:** 10.1038/ncomms10897

**Published:** 2016-03-30

**Authors:** D. L. Campbell, R. M. Price, A. Putra, A. Valdés-Curiel, D. Trypogeorgos, I. B. Spielman

**Affiliations:** 1 Joint Quantum Institute, University of Maryland, College Park, Maryland 20742, USA

## Abstract

Phases of matter are characterized by order parameters describing the type and degree of order in a system. Here we experimentally explore the magnetic phases present in a near-zero temperature spin-1 spin–orbit-coupled atomic Bose gas and the quantum phase transitions between these phases. We observe ferromagnetic and unpolarized phases, which are stabilized by spin–orbit coupling's explicit locking between spin and motion. These phases are separated by a critical curve containing both first- and second-order transitions joined at a tricritical point. The first-order transition, with observed width as small as *h* × 4 Hz, gives rise to long-lived metastable states. These measurements are all in agreement with theory.

## Abstract (web summary)

Phases of matter are characterized by order parameters describing the type and degree of order in a system. Here, the authors explore the magnetic phases and the phase transitions present in a near-zero temperature spin-1 spin-orbit-coupled Bose gas, observing ferromagnetic and unpolarized states.

## Graphical abstract

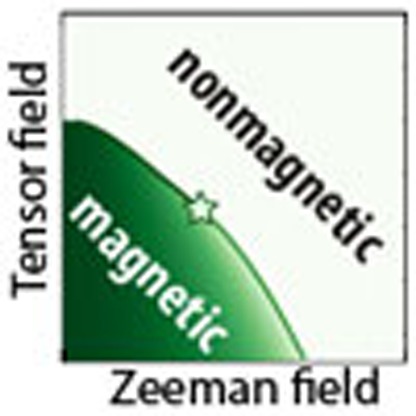

Most magnetic systems are composed of localized particles such as electrons^1^, atomic nuclei^2^ and ultracold atoms in optical lattices^3^,^4^,^5^,^6^, each with a magnetic moment *μ*. By contrast, itinerant magnetism^7^,^8^ describes systems where the magnetic particles, here ultracold neutral atoms, can themselves move freely, and for which magnetism is generally weak. Our spin–orbit-coupled Bose–Einstein condensates^9^,^10^,^11^,^12^ (BECs) constitute a magnetically ordered itinerant system in which—unlike more conventional spinor BECs^13^—the atoms' kinetic energy explicitly drives a phase transition between two different ordered phases^10^. While the coupling between spin and momentum afforded by spin–orbit coupling (SOC) is insufficient to stabilize ferromagnetism in itinerant fermionic systems^14^, in bosonic systems it leads to magnetic phases that are not present in spinor BECs without SOC^11^,^12^.

Phase transitions can generally be described in terms of a free energy *G*(*M*_*z*_)—including the total internal energy along with thermodynamic contributions that are negligible for our nearly zero temperature *T*=0 system—that is minimized for an equilibrium system. Here the magnetization 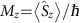 is an order parameter, associated with the spin 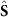, which changes abruptly as our system undergoes a phase transition. Figure 1c shows typical *T*=0 free energies: a first-order phase transition (top panel) occurs when the number of local minima in *G*(*M*_*z*_) stays fixed, but the identity of the global minima changes; and a second-order phase transition (bottom panel) occurs when degenerate global minima merge or separate. These defining features are true both for *T*>0 thermal and *T*=0 quantum phase transitions.

For spin-1/2 systems (that is, total angular momentum, *f*=1/2) like electrons, ferromagnetic order can be represented in terms of a magnetization vector 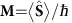. This is rooted in the fact that the three components of the spin operator 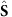 transform vectorially under rotation. More specifically, any Hamiltonian describing a two level system may be expressed as 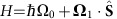, the sum of a scalar (rank-0 tensor) and a vector (rank-1 tensor) contribution. The former, described by Ω_0_ gives an overall energy shift, and the latter takes the form of the linear Zeeman effect from an effective magnetic field proportional to **Ω**_1_. Going beyond this, fully representing a spin-1 (total angular momentum *f*=1 with three *m*_F_ sublevels: |−1〉, |0〉, and |+1〉) Hamiltonian with angular momentum 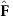 requires an additional five-component rank-2 tensor operator—the quadrupole tensor—and therefore there exist ‘magnetization' order parameters that are not simply associated with any spatial direction^13^,^15^,^16^.

Pioneering studies in GaAs quantum wells^17^,^18^ showed that material systems with equal contributions of Rashba and Dresselhaus SOC described by the term 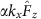, subject to a transverse magnetic field with Zeeman term 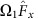, can equivalently be described as a spatially periodic effective magnetic field. Our experiments with spin-1 atomic systems use ‘Raman' lasers with wavelength *λ* to induce SOC of this form^9^,^19^,^20^,^21^,^22^,^23^,^24^ with strength *α*=2*ħk*_R_/*m*, where the single-photon recoil energy and momentum are 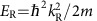 and *ħk*_R_=2*πħ*/*λ*. This atomic system can therefore be described by the magnetic Hamiltonian


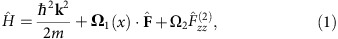


describing atoms with mass *m* and momentum *ħ***k** interacting with an effective Zeeman magnetic field **Ω**_1_(*x*)/Ω_1_=cos(2*k*_R_*x*)**e**_*x*_−sin(2*k*_R_*x*)**e**_*y*_ helically precessing in the **e**_*x*_−**e**_*y*_; and an additional Zeeman-like tensor coupling with strength Ω_2_. Here, 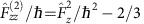 is an element of the quadrupole tensor operator. The competing contributions between kinetic and magnetic ordering energies (interactions select between different nearly degenerate ground states, but only weakly perturb the location of the phase transitions, see Methods) make ours an archetype system for studying exotic magnetic order and understanding the associated quantum phase transitions, of which both first and second order are present in our experiments (Fig. 1b).

We can easily understand the first-order transition in the limit of infinitesimal Ω_1_ where the tensor field favours either: a polar BEC for Ω_2_>0 (*m*_F_=0: unmagnetized, *M*_*z*_=0), or a ferromagnetic BEC for Ω_2_<0 (*m*_F_=+1 or −1: magnetized, |*M*_*z*_|=1). As with spinor BECs^25^, these phases are separated by a first-order phase transition at Ω_2_=0. The ferromagnetic phase spontaneously breaks the Z_2_ symmetry associated with the Hamiltonian's invariance under the exchange |−1〉↔|+1〉. The second-order transition can be intuitively understood by considering the large Ω_1_ limit where the system forms a spin–helix BEC (with local magnetization antiparallel to **Ω**_1_: unmagnetized, *M*_*z*_=0). This order increases the system's kinetic energy, leading to the second-order phase transition into the ferromagnetic phase shown in Fig. 1b as Ω_1_ decreases. Analogues to this second-order phase transition are present in other systems with effective spin degrees of freedom such as double-leg ladders^26^ or engineered optical lattices^27^,^28^.

These two extreme limits continuously connect at the point 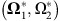, the green star in Fig. 1b, where the small-Ω_1_ first-order phase transition gives way to the large-Ω_1_ second-order transition, and together these regions constitute a curve of critical points 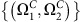. Here we realized spin-1 spin–orbit-coupled BECs and varied the magnetic coupling fields using externally applied fields. By directly measuring the system's magnetization, we studied the associated quantum phase transitions present in the phase diagram, all in quantitative agreement with theory.

## Results

### Experimental set-up

**Figure 1:**
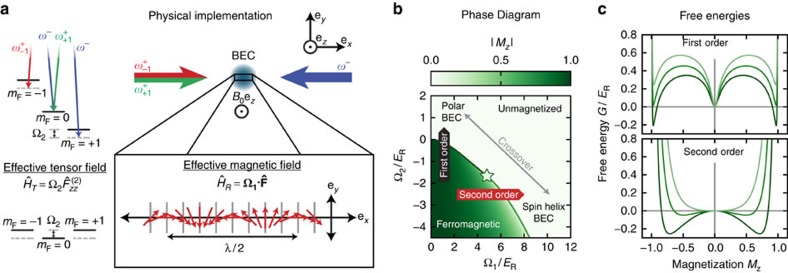
Experimental system. (**a**) Schematic and level diagram. The |−1〉↔|0〉 and |0〉↔|+1〉 transitions of the *f*=1 ground state manifold of ^87^Rb were independently Raman coupled, giving experimental control of Ω_1_ and Ω_2_. (**b**) Phase diagram. The ferromagnetic order parameter |*M*_*z*_| is plotted against Ω_2_ and Ω_1_. The solid (dashed) red curve denotes the first-order (second-order) transition from the magnetized phase. (**c**) Free energies. Top: near the first-order phase transition at Ω_1_/*E*_R_=1 for Ω_2_/*E*_R_=−0.35, −0.1 and 0.15 for the black, blue and red traces respectively, as marked by the red flags in **b**. Bottom: near the second-order phase transition at Ω_2_/*E*_R_=−2.5 for Ω_1_/*E*_R_=4.5, 5.5, and 6.5 for the black, blue and red traces, respectively.

As shown in Fig. 1a, we realized this magnetic system by illuminating ^87^Rb BECs in the *f*=1 ground state manifold with a pair of counter propagating and orthogonally polarized Raman lasers that coherently coupled the manifold's *m*_F_ states. Physically, the spatial interference of the orthogonally polarized laser beams give rise to the helical effective magnetic field (see Methods) with period *λ*/2. As we first showed^9^ using effective *f*=1/2 systems, this introduces both a spin–orbit and a Zeeman term into the BEC's Hamiltonian, equivalent to equation (1). Here the quadratic Zeeman shift from a large bias magnetic field *B*_0_e_*z*_ split the low-field degeneracy of the |−1〉↔|0〉 and |0〉↔|+1〉 transitions, and we independently Raman coupled these state pairs with equal strength Ω_1_. We dynamically tuned the quadrupole tensor field strength Ω_2_ by simultaneously adjusting the Raman frequency differences; as shown in Fig. 1 we selected frequencies differences where the detuning from the |+1〉 to |0〉 and |−1〉 to |0〉 were both equal to Ω_2_ (see Methods). Without this technique, only the upper half plane of the phase diagram (Fig. 1b) would be accessible: containing only an unmagnetized phase, therefore lacking any phase transitions.

In each experiment, we first prepared BECs at a desired point in the phase diagram, possibly having crossed the phase transition during preparation. A combination of trap dynamics^29^,^30^, collisions and evaporation^31^ kept the system in or near (local) thermal equilibrium. We then made magnetization measurements directly from the Bose-condensed atoms measured in the spin resolved momentum distribution obtained using the time-of-flight (TOF) techniques described in ref. 30.

### Critical line of phase transitions

**Figure 2:**
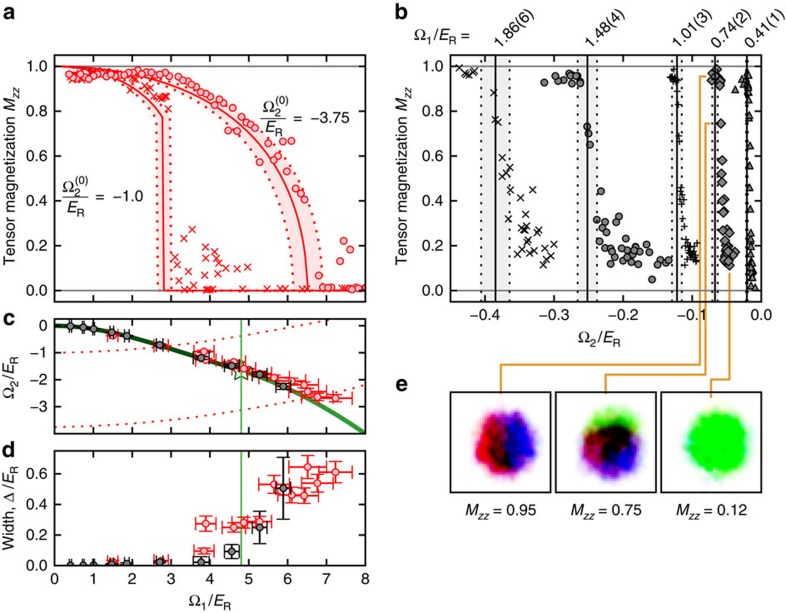
Measured phase transition. (**a**) Tensor magnetization *M*_*zz*_ measured as a function of Ω_1_, showing both second-order [Ω_2_(Ω_1_=0)=−3.7500(3)*E*_R_] and first-order [Ω_2_(Ω_1_=0)=−1.0*E*_R_] phase transitions in comparison with theory. These curves followed the nominally horizontal trajectories (see Methods) marked by red dashed curves in **c**. (**b**) Tensor magnetization measured as a function of Ω_2_ at Ω_1_/*E*_R_=1.86(6), 1.48(4), 1.01(3), 0.74(2), and 0.41(1), plotted along with the predicted critical Ω_2_. In **a**,**b** the light-coloured region reflects the uncertainty in theory resulting from our ≈5% systematic uncertainty in Ω_1_. (**c**,**d**) Phase transition. Black (red) symbols depict data obtained using vertical (nominally horizontal) cuts through the phase diagram. (**c**) measured phase transitions plotted along with theory: solid (phase transition), and green vertical line (tricritical point, 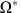) Horizontal error bars correspond to one s.d. on Ω_1_ and vertical error bars are the 95% confidence intervals from the fitting function that determines the critical point. (**d**) 20 to 50% transition width showing the clear shift from first- to second-order with increasing Ω_1_. (**e**) Domain formation for Ω_1_=0.74(2) showing interaction-driven spin structure near the first-order phase transition. In all images, red corresponds to spatial regions with local *M*_*z*_≈−1, the green regions correspond to *M*_*z*_≈0 and the blue regions correspond to *M*_*z*_≈+1. In the polar regime at *M*_*zz*_=0.12 only the *m*_F_=0 cloud is visible; near the first-order phase transition at *M*_*zz*_=0.75 all three *m*_F_=0 clouds are visible and have partially phase separated; and in the ferromagnetic regime at *M*_*zz*_=0.95 only *m*_F_±1 clouds are visible and they have completely phase separated.

Our experiment first focused on thermodynamic phase transitions. We made vertical (horizontal) scans through the phase diagram by initializing the system in the unmagnetized phase at a desired value of Ω_1_ (Ω_2_) with Ω_2_≳;0 (Ω_1_≲10*E*_R_), and then ramping Ω_2_ (Ω_1_) through the transition region. (As discussed in the methods our nominally horizontal scans of Ω_1_ followed slightly curved trajectories through the phase diagram, such as the red dashed curve in Fig. 2c). Following such ramps, domains with both ±*M*_*z*_ can rapidly form, and we therefore focus on the tensor magnetization 
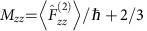
, which is sensitive to this local magnetic order.

Using horizontal scans, we crossed through the second-order phase transition 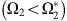 where the free energy evolves continuously from having one minimum (with *M*_*zz*_=0, for large Ω_1_) to having two degenerate minima (with *M*_*zz*_>0, for smaller Ω_1_). As shown in Fig. 2a, *M*_*zz*_ continuously increases with decreasing Ω_1_, reaching its saturation value as Ω_1_→0. Repeating these processes for 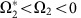, we found a sharp first-order transition. In each case, data are plotted along with theory with no adjustable parameters. Using data of this type for a range of Ω_2_ and fitting to numeric solutions of equation (1), we obtained the critical points plotted in Fig. 2c. Because horizontal cuts through the phase diagram are nearly tangent to the transition curve for small Ω_2_, this produced large uncertainties in 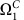 for the first-order phase transition.

We studied the first-order phase transition with greater precision by ramping Ω_2_ through the transition at fixed Ω_1_ (Fig. 2b) and found near perfect agreement with theory. For all the experimentally measured critical points, see Fig. 2c top, separating the unmagnetized and ferromagnetic phase, we also measured the corresponding transition width defined as the required interval for the curve to fall from 50 to 20% of its full range. This width Δ decreases sharply at 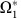, marking the crossover between second- and first-order phase transitions (see Fig. 2c bottom). In these data, the width of the first-order transition becomes astonishingly narrow: as small as 0.0011(3)*E*_R_=*h* × 4(1) H*z* at Ω_1_=0.41(1). This narrowness results from the energetic penalty associated with condensation into multiple modes for repulsively interacting bosons. In addition we find that ramps through the first-order transition are hysteretic, and very slow ramps (see Methods) for the system to equilibrate.

During the long equilibration times required to study this transition, spin-domains formed in our system, shown in Fig. 2e. For Bose-condensed atoms, our TOF procedure expanded the initial spin-distribution allowing us to reconstruct any *in situ* spin structure. Figure 2e shows that domains form as the systems enters into the magnetized phase; these domains mark the role of interactions in spontaneously breaking the Hamiltonian's *Z*_2_ symmetry (see Methods for a comparison with sodium where the sign of the spin-dependent interactions is reversed, and the *Z*_2_ symmetry remains unbroken for a wide range of parameters). Figure 2b shows that additional tripartite spin structure is present very near the first-order phase transition, which was not anticipated in our initial single-particle description. This tripartite mixture, predicted in ref. 32 is an in-plane ferromagnetic phase with no analogue in spinor BECs or effective spin-1/2 SOC BECs.

### Metastable states

**Figure 3:**
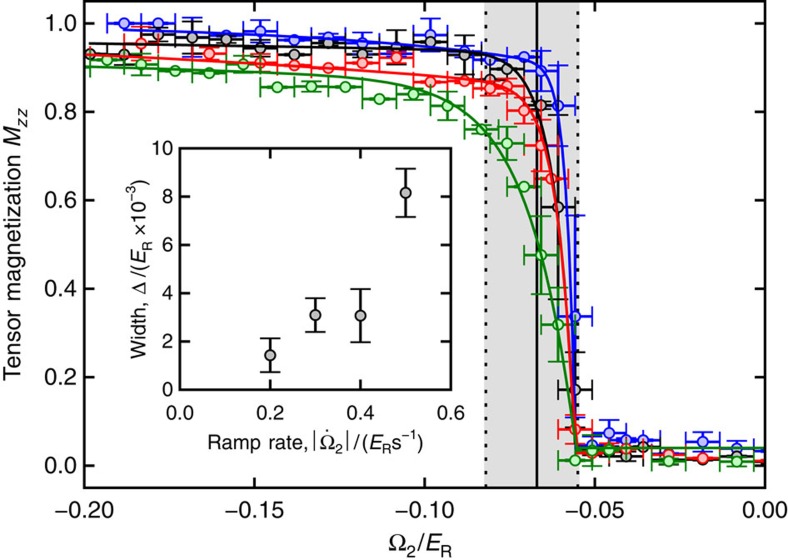
Quenching dynamics. The system was prepared in the unmagnetized phase with Ω_1_=0.74(8)*E*_R_ and Ω_2_ was ramped through the phase transition at ramp-rates dΩ_2_/d*t*=−0.2, −0.3, −0.4, and −0.5*E*_*R*_ s^−1^ (blue, black, red, and green symbols, respectively). The curves are guides to the eye. The inset shows the decreasing width, defined as the required interval for the curve to fall from 50 to 20% of its full range, of the first-order transition as the ramp-rate decreases. Vertical error bars correspond to one standard deviation of up to 10 measurements, and horizontal error bars correspond to a systematic error of 0.005*E*_R_ from fluctuations in the bias field. Error bars on the inset panel correspond to the 95% confidence interval on the fitting function of the quenching data.

We observed that scans crossing the second-order transition typically required 50 ms to equilibrate, while for scans crossing the first-order transitions we allowed as long as 1.5 s for equilibration. Systems taken through a first-order phase transition can remain in long-lived metastable states. Here a metastable state with *M*_*z*_=0 persists in the ferromagnetic phase, and a pair of metastable states with *M*_*z*_≠0 persists in the unmagnetized phase. We began our study of this metastability by quenching through the first-order transition at Ω_1_=0.74(8)*ER* with differing rates from −0.5 to −0.2 *E*_R_ s^−1^, as shown in Fig. 3. We observed the transition width continuously decreases with decreasing absolute value ramp rate (inset to Fig. 3), consistent with slow relaxation from a metastable initial state.

**Figure 4:**
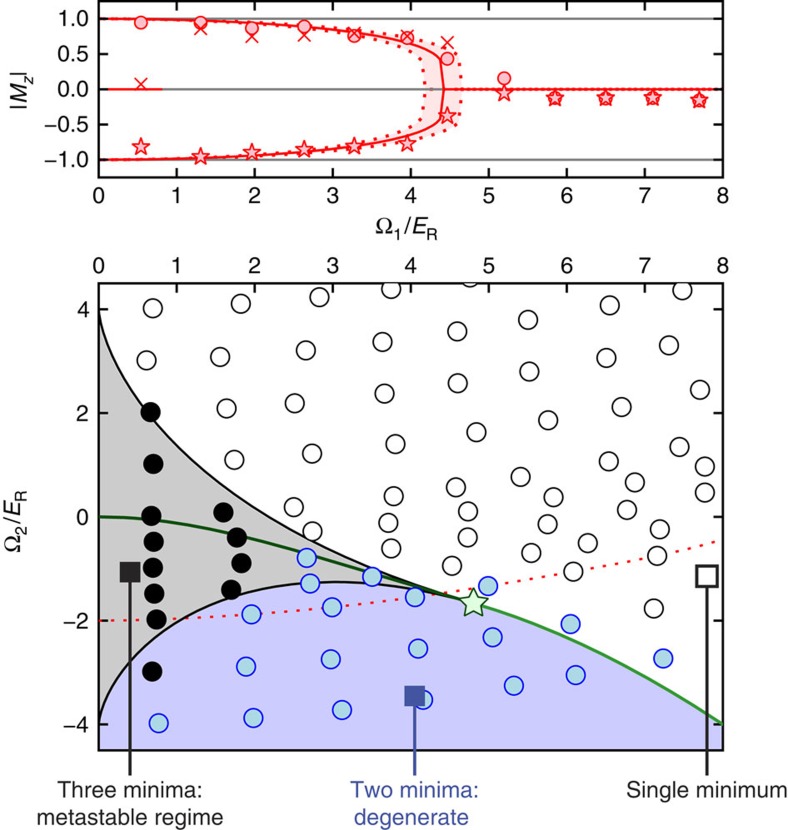
Metastable states. Top, Measured magnetization plotted along with theory. The system was prepared at the desired Ω_2_=−2*E*_R_; Ω_1_(*t*) was then increased to its displayed final value; during this ramp Ω_2_ also changed, and the system followed the curved trajectory in the bottom panel. Each displayed data point is an average of up to 10 measurements, and the coloured region reflects the uncertainty in theory resulting from our ≈5% systematic uncertainty in Ω_1_. Circles/crosses/stars represent data starting in *m*_F_=+1, 0, and −1 respectively. Bottom, state diagram: theory and experiment. Blue: two states; black: three states; white: one state. Coloured areas denote calculated regions where the colour-coded number of stable/metastable states are expected. Symbols are the outcome of experiment. Each displayed data point is an average of up to 20 measurements.

We explored the full regime of metastability by initializing BECs in each of the *m*_F_ states, at fixed Ω_2_, then rapidly ramping Ω_1_(*t*) from zero to its final value fast enough that the system did not adiabatically follow into the true ground state, yet slow enough that the quasi-equilibrium metastable state was left near its local equilibrium. We found that the rate ≲200*E*_R_ *s*^−1^ was a good compromise between these two requirements. For points near the first-order phase transition three metastable states exist (Fig. 4); near the second-order transition this count decreases, giving two local minima which merge to a single minimum beyond the second-order transition.

We experimentally identified the number of metastable states by using *M*_*z*_ and its higher moments, having started in each of the three *m*_F_ initial states. A small variance in *M*_*z*_, <0.25, indicates the final states are clustered together—associated with a single global minimum in the free energy *G*(*M*_*z*_)—and it increases when metastable or degenerate ground states are present. We distinguished systems with two degenerate magnetization states (*M*_*z*_≈±1) from those with three states by the same method, since when *M*_*z*_≈±1, the variance of |*M*_*z*_| is smaller than 0.25, and it distinguishably increases beyond 0.25 as a third metastable state appears with *M*_*z*_=0. In this way we fully mapped the system's metastable states in agreement with theory, as shown in Fig. 4

## Discussion

We accurately measured the two-parameter phase diagram of a spin-1 BEC, containing a ferromagnetic phase and an unmagnetized phase, continuously connecting a polar spinor BEC to a spin–helix BEC. The ferromagnetic phase in this itinerant system is stabilized by SOC, and vanishes as the SOC strength *ħk*_R_ goes to zero. Our observation of controlled quench dynamics through a first-order phase transition opens the door for realizing Kibble–Zurek physics^33^,^34^ in this system, where the relevant parameters can be controlled at the individual Hz level. The quadrupole tensor field 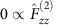 studied here is the *q*=0 component of the rank-2 spherical tensor operator 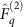, with *q*∈{±2, ±1, 0}. The physics of this system would be further enriched by the addition of the remaining four tensor fields. The *q*=0 term we included is the most simple of the tensor fields to deploy, as it only required control over frequencies. The *q*=±1 components are relatively simple to incorporate by radio frequency (RF)-coupling the |*m*_F_=−1〉 to |*m*_F_=0〉 and |*m*_F_=+1〉 to |*m*_F_=0〉 transitions with different phases. The *q*=±2 components require direct coupling between |*m*_F_=+1〉 and |*m*_F_=−1〉, which is straightforward using two-photon microwave transitions, but is challenging to include with significant strength.

## Methods

### System preparation

We created *N*≈4 × 10^5^ atoms ^87^Rb BECs in the ground electronic state |*f*=1〉 manifold^35^, confined in the locally harmonic trapping potential formed at the intersection of two 1,064-nm laser beams propagating along **e**_*x*_ and **e**_*y*_ giving trap frequencies of (*ω*_*x*_, *ω*_*y*_, *ω*_*z*_)/2*π*=(33(2), 33(2), 145(5)) Hz. The quadratic contribution to the *B*_0_=35.468(1) G magnetic field's ≈*h* × 25 MHz Zeeman shift lifts the degeneracy between the |*f*=1, *m*_F_=−1〉↔|*f*=1, *m*_F_=0〉 and |*f*=1, *m*_F_=0〉↔|*f*=1, *m*_F_=1〉 transitions, by 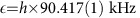. We denote the energy differences between these states as *ħδω*_−1,0_, *ħδω*_0,+1_ and *ħδω*_−1,+1_.

### Frequency selective Raman coupling

We Raman-coupled the three *m*_F_ states using a pair of *λ*=790.024(5) nm laser beams counter-propagating along **e**_*x*_. The beam travelling along +**e**_*x*_ had frequency components 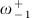 and 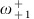, while the beam travelling along −**e**_*x*_ contained the single frequency *ω*^−^. These beams were linearly polarized along **e**_*z*_ and **e**_*y*_, respectively. The frequencies were chosen such that the differences 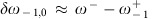 and 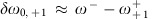 independently Raman coupled the |*m*_F_=−1, 0〉 and |*m*_F_=0, +1〉 state-pairs, respectively. Furthermore we selected 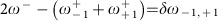 such that, after making the rotating wave approximation (RWA) the |*m*_F_=±1〉 states were energetically shifted by the same 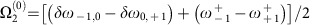 energy from |*m*_F_=0〉, thereby yielding the frequency-tuned tensor energy shift depicted in Fig. 1a. In addition, Ω_2_ in equation (1) differs from 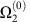 by a small shift 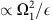 resulting from off-resonant coupling to transitions detuned by 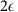, which we computed directly using Floquet theory (see equation (6) in Methods). This tensor contribution 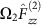 to the Hamiltonian might equivalently be introduced by a quadratic Zeeman shift alone, giving 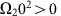.

### The physical basis for SOC using Raman lasers

As explained in ref. 36, the spin-dependent (vector) part of the light–matter interaction can be written in terms of an effective Zeeman field


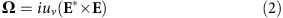


with overall strength given by *u*_*v*_ as defined in ref. 36, where **E** is the total optical electric field from the Raman lasers. This field then enters into the Hamiltonian as an effective magnetic field


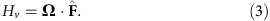


The electric field for the laser geometry depicted in Fig. 1a is 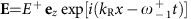

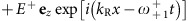
+*iE*^−^**e**_*y*_ exp[*i*(−*k*_R_*x*−*ω*^−^*t*)], giving rise to the time-dependent effective Zeeman coupling term


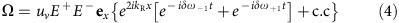



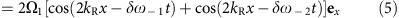


where we defined 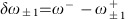. This gives the Hamiltonian term


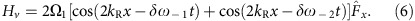


In our experiment the *ω*_*Z*_/2*π* ≈25 MHz linear Zeeman shift is large compared with all other energy scales, so we make the RWA to arrive at the low-frequency Hamiltonian


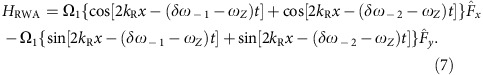


We selected our frequencies to be in four-photon resonance with the |−1〉 to |+1〉 transition, giving (*δω*_−1_−*ω*_*Z*_)=−(*δω*_+1_−*ω*_*Z*_), in which case the Hamiltonian is time-periodic. Equation (1) in the manuscript is obtained by making independent RWAs on the |−1〉→|0〉 and |0〉→|+1〉 transitions separately, giving the helically precessing coupling described in the manuscript.

### Floquet and polynomial shift

The frequency differences 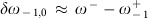 and 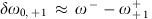 nominally Raman dress both |−1, 0〉 and |0, +1〉 state pairs independently. In practice, the cross coupling may be substantial and the adjusted eigenenergies may be computed exactly from Floquet theory. For our 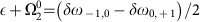 separation between Floquet bands (in our experiment 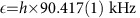) we find the relationship between Ω_1_, Ω_2_ and 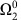 is well described by the polynomial


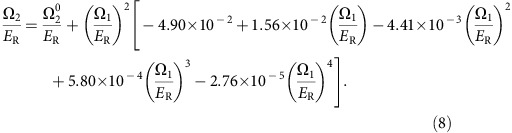


### Measurement details

In the horizontal scans in Fig. 1 of the manuscript, we ramped Ω_1_ at a rate of ≈−40*E*_R_/s, allowing the system to adiabatically track the ground state, and allowed 50 ms for equilibration before the measurement process. In contrast, for our vertical scans we found the system required between 0.2 ms and 2 s to equilibrate.

We studied the metastable states by performing three separate experiments for each raw data point: one each for a system initially prepared in *m*_F_=0, ±1 state at the desired value of Ω_2_. We then adiabatically ramped Ω_1_ from zero to its final value. For each resulting (Ω_1_, Ω_2_) pair, we obtained the magnetization *M*_*z*_ for each initial state. In all of the described procedures Ω_1_ was turned off immediately, at the start of our 28 ms time-of-flight imaging process, which included a Stern–Gerlach gradient to separate the spin components.

### Operators

The total angular momentum *f*=1 spin operators in equation (1) of the main manuscript take the explicit form


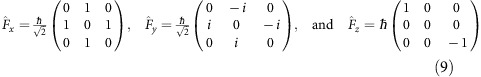


in the basis of the magnetic sublevels |−1〉, |0〉, and |+1〉; together these comprise the vector operator 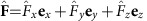. Likewise the quadrupole tensor operator is expressed as


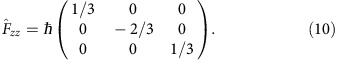


In terms of these operators it is clear than any Hamiltonian involving only 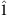, 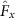, 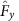 and 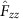 is invariant under the transformation that swaps |+1〉 and |−1〉: a discrete Z_2_ symmetry.

### Wavefunctions

The wavefunctions in the polarized and unpolarized regimes are qualitatively different. In the unpolarized regime the wavefunction takes the general form 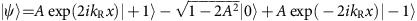. The value of *A* depends in detail on Ω_1_ and Ω_2_, but two limits are clear. First, when Ω_1_=0 and Ω_2_>0 the system forms a spinor BEC in the polar phase, with *A*=0: a BEC in *m*_F_=0. Second, for Ω_1_→∞ the local spin follows Ω giving 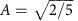.

In the polarized regime, the wavefunction has the general form 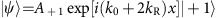 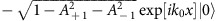 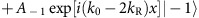, but with constraints: first, 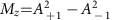 (the definition of magnetization); and second, *M*_*z*_=−*k*_0_/2*k*_R_ (ensuring zero center-of-mass motion, see ref. 30). In our experiment *m*_F_=±1 are coupled at second order in Ω_1_, and for 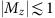, these states are ≈16*E*_R_ detuned. Thus, for *M*_*z*_≲+1, we have *A*_−1_≈0 with corrections at order 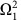, giving the wavefunction 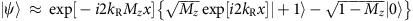 in terms of the magnetization.

### Free energy and phase diagram

We obtained the free energy *G*(*M*_*z*_) as a function of the magnetization *M*_*z*_ by first numerically solving the system's Hamiltonian given by equation (1), obtaining the eigenenergies *E*_*σ*_(**k**) and state 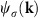, each identified by a momentum *ħ***k** and a ‘band' index *σ*∈{−1, 0, +1}. We then computed *M*_*z*_ for each of these states (dependent on *k*_*x*_, but independent of *k*_*y*_ and *k*_*z*_), thereby obtaining the internal energy *E*(*M*_*z*_) in the lowest band (*σ*=−1).

As our BEC is very near the ground state the free energy *G*(*M*_*z*_)=*E*(*M*_*z*_)−*TS*, where *T* is the temperature and *S* is the entropy is well approximated by *G*(*M*_*z*_)≈*E*(*M*_*z*_), and it is this free energy, which is plotted in Fig. 1c. We then obtained the phase diagram in Fig. 1b by numerically computing the free energy for each pair Ω_1_, Ω_2_ and identifying its equilibrium magnetization.

For non-interacting systems, we found that the curves defining the phase transitions and also those bounding the region containing metastable states could all be computed in closed form. First, the critical point at which the first- and second-order phase transitions meet is at


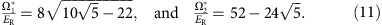


The curve defining the first-order phase transition (for 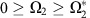) is given by


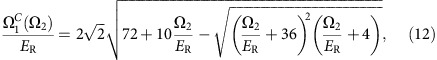


and the curve defining the second-order phase transition (for 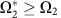) is given by


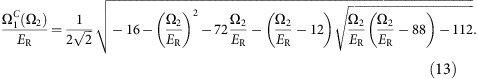


The upper boundary of the metastable regime (in the unmagnetized phase) is given by


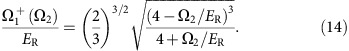


and the lower boundary of the metastable regime (in the ferromagnetic phase) is given by


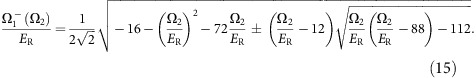


This is the same equation defining the second-order phase transition with an added ±, the full curve defining the boundary of the metastable regime crosses over between the + and − solutions at 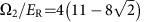.

### Magnetic fields

Because the free energy *G*(*M*_*z*_) is sensitive to unwanted detuning *δ* from the four-photon resonance near the phase transitions, which contributes an added symmetry breaking field 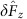 to the Hamiltonian, controlling the bias magnetic field and nulling its gradients is critical. A pair of flux-gate sensors measuring the ambient magnetic field along **e**_*z*_, allowed us to compensate for long-term field drifts. We compensated any field gradients using four pairs of anti-Helmholtz coils in a clover leaf configuration^37^, and a conventional anti-Helmholtz pair, all aligned along **e**_*z*_.

### Interactions

We studied the impact of interactions at the level of mean field theory using a variational approach, assuming an infinite homogenous system. For each point in the phase diagram labelled by (Ω_1_, Ω_2_), we first located the local minima in the single-particle free energy described by the Hamiltonian 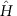 of equation (1) in the manuscript. The free energy had from one to three minima, with energies *E*_*j*_ and eigenstates 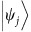.

We then considered an infinite system and minimized the mean field energy density


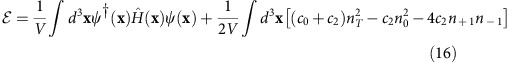


for an arbitrary linear combination of these single particle states with amplitudes *α*_*j*_, where *n*_*σ*_(**x**) is the local density in a given spin state *σ*; *n*_*T*_(**x**) is the total local density; and *c*_0_ and *c*_2_ are the spin-independent and spin-dependent interactions, respectively. For ^87^Rb87 these have the ratio *c*_2_/*c*_0_≈−0.005 and for ^23^Na they are *c*_2_/*c*_0_≈+0.05. In our minimization, we modelled our systems with a typical mean-field energy of (*c*_0_+*c*_2_)*n*_*T*_≈1 kHz per particle.

**Figure 5:**
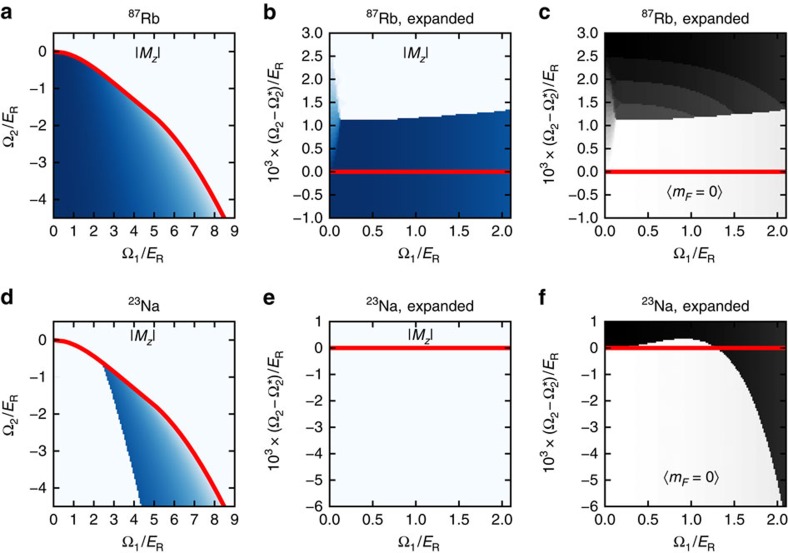
Mean field phase diagram. (**a**–**c**) Rubidium. (**d**–**f**) Sodium. In each panel, the red curve marks the location of the phase transition as computed excluding interactions. (**b**,**c**,**e**,**f**) Expanded views of the region around the critical curve. **a**,**b**,**d** and **e** plot the |*M*_*z*_| order parameter: the plot is dark blue when |*M*_*z*_|=1 and there is a continuous gradation to white when |*M*_*z*_|=0. **c** and **f** plot the fraction of the wavefunction in *m*_F_=0: similarly, black colour indicates when all the atoms are in *m*_F_=0 and white when none of the atoms are in *m*_F_=0.

Figure 5 shows the result of this calculation both for rubidium and sodium. In both cases the overall phase diagram (Fig. 5a,d) is shaped by the single-particle Hamiltonian; at this coarse level the rubidium phase diagram is hardly different from that predicted from single particle physics, but in the case of sodium a large swath of the expected ferromagnetic phase remains symmetry unbroken. This phase continuously connects to an equal superposition of |−1〉 and |+1〉 as Ω_1_→0.

The situation becomes more complex as we focus on *M*_*z*_ near the curve defining the first-order phase transition (Fig. 5b,e), for the case of rubidium a new mixed phase appears at low Ω_1_ analogous to the striped phase in spin−1/2 systems, but nothing new is apparent for sodium.

Last, we consider the same region, but looking at the fraction of the variational wavefunction in the *m*_F_=0 spin state. For rubidium, this allows us to identify a new state which is a three-way mixture of all three components considered in the variational calculation (with no analogue in the Ω_1_=0 spinor limit), and we can see the abrupt transition in sodium from a state connecting to the polar phase (Ω_2_>0) and to the uniaxial nematic phase (Ω_2_<0). Each of these phases are as described in ref. 32.

## Additional information

**How to cite this article:** Campbell, D. L. *et al.* Magnetic phases of spin-1 spin–orbit-coupled Bose gases. *Nat. Commun.* 7:10897 doi: 10.1038/ncomms10897 (2016).

## References

[b1] Aharoni A. Introduction to the Theory of Ferromagnetism Oxford Univ. Press (2011) .

[b2] Abragam A. Nuclear ferromagnetism and ant ferromagnetism. Contemp. Phys. 33, 305–312 (1992) .

[b3] Kronjäger J., Becker C., Soltan-Panahi P., Bongs K. & Sengstock K. Spontaneous pattern formation in an antiferromagnetic quantum gas. Phys. Rev. Lett. 105, 090402 (2010) . 20868141 10.1103/PhysRevLett.105.090402

[b4] Simon J. *et al.* Quantum simulation of antiferromagnetic spin chains in an optical lattice. Nature 472, 307–312 (2011) . 21490600 10.1038/nature09994

[b5] Greif D., Uehlinger T., Jotzu G., Tarruell L. & Esslinger T. Short-range quantum magnetism of ultracold fermions in an optical lattice. Science 240, 1307–1310 (2013) . 23704375 10.1126/science.1236362

[b6] Hart R. A. *et al.* Observation of antiferromagnetic correlations in the hubbard model with ultracold atoms. Nature 519, 211–214 (2015) . 25707803 10.1038/nature14223

[b7] Stoner E. Atomic moments in ferromagnetic alloys with non- ferromagnetic elements. Philos. Mag. 15, 1018–1034 (1933) .

[b8] Sanner C. *et al.* Correlations and pair formation in a repulsively interacting Fermi gas. Phys. Rev. Lett. 108, 240404 (2012) . 23004242 10.1103/PhysRevLett.108.240404

[b9] Lin Y.-J., Jiménez-Garca K. & Spielman I. B. Spin-orbit-coupled Bose-Einstein condensates. Nature 471, 83–86 (2011) . 21368828 10.1038/nature09887 PMC11493149

[b10] Lan Z. & Öhberg P. Raman-dressed spin-1 spin-orbit-coupled quantum gas. Phys. Rev. A 89, 023630 (2014) .

[b11] Radić J., Natu S. S. & Galitski V. Stoner ferromagnetism in a thermal pseudospin-1/2 Bose gas. Phys. Rev. Lett. 113, 185302 (2014) . 25396377 10.1103/PhysRevLett.113.185302

[b12] Hickey C. & Paramekanti A. Thermal phase transitions of strongly correlated bosons with spin-orbit coupling. Phys. Rev. Lett. 113, 265302 (2014) . 25615348 10.1103/PhysRevLett.113.265302

[b13] Stamper-Kurn D. M. & Ueda M. Spinor Bose gases: symmetries, magnetism, and quantum dynamics. Rev. Mod. Phys. 85, 1191–1244 (2013) .

[b14] Zhang S.-S., Ye J. & Liu W.-M. Itinerant ferromagnetism in repulsively interacting spin-orbit coupled Fermi gas. Preprint at http://arxiv.org/abs/1403.7031 (2014) .

[b15] Stenger J. *et al.* Spin domains in ground-state bose-einstein condensates. Nature 396, 345–348 (1998) .

[b16] Barnett R., Turner A. & Demler E. Classifying Novel Phases of Spinor Atoms. Phys. Rev. Lett. 97, 180412 (2006) . 17155529 10.1103/PhysRevLett.97.180412

[b17] Stanescu T. D. & Galitski V. Spin relaxation in a generic two-dimensional spin-orbit coupled system. Phys. Rev. B 75, 125307 (2007) .

[b18] Koralek J. D. *et al.* Emergence of the persistent spin helix in semiconductor quantum wells. Nature 458, 610–613 (2009) . 19340077 10.1038/nature07871

[b19] Wang C., Gao C., Jian C.-M. & Zhai H. Spin-orbit coupled spinor bose-einstein condensates. Phys. Rev. Lett. 105, 160403 (2010) . 21230952 10.1103/PhysRevLett.105.160403

[b20] Ho T.-L. & Zhang S. Bose-Einstein condensates with spin-orbit interaction. Phys. Rev. Lett. 107, 150403 (2011) . 22107273 10.1103/PhysRevLett.107.150403

[b21] Zhang J.-Y. *et al.* Collective dipole oscillations of a spin-orbit coupled Bose-Einstein condensate. Phys. Rev. Lett. 109, 115301 (2012) . 23005641 10.1103/PhysRevLett.109.115301

[b22] Wang P. *et al.* Spin-orbit coupled degenerate Fermi gases. Phys. Rev. Lett. 109, 095301 (2012) . 23002843 10.1103/PhysRevLett.109.095301

[b23] Cheuk L. *et al.* Spin-injection spectroscopy of a spin-orbit coupled Fermi gas. Phys. Rev. Lett. 109, 095302 (2012) . 23002844 10.1103/PhysRevLett.109.095302

[b24] Galitski V. & Spielman I. B. Spin-orbit coupling in quantum gases. Nature 494, 49–54 (2013) . 23389539 10.1038/nature11841

[b25] Sadler L. E., Higbie J. M., Leslie S. R., Vengalattore M. & Stamper-Kurn D. M. Spontaneous symmetry breaking in a quenched ferromagnetic spinor Bose-Einstein condensate. Nature 443, 312–315 (2006) . 16988706 10.1038/nature05094

[b26] Atala M. *et al.* Observation of chiral currents with ultracold atoms in bosonic ladders. Nat. Phys. 10, 588–593 (2014) .

[b27] Struck J. *et al.* Quantum simulation of frustrated classical magnetism in triangular optical lattices. Science 333, 996–999 (2011) . 21778359 10.1126/science.1207239

[b28] Parker C. V., Ha L.-C. & Chin C. Direct observation of effective ferromagnetic domains of cold atoms in a shaken optical lattice. Nat. Phys. 9, 769–774 (2013) .

[b29] Lin Y.-J. *et al.* Bose-Einstein condensate in a uniform light-induced vector potential. Phys. Rev. Lett. 102, 130401 (2009) . 19392335 10.1103/PhysRevLett.102.130401

[b30] Lin Y.-J. *et al.* A synthetic electric force acting on neutral atoms. Nat. Phys. 7, 531–534 (2011) .

[b31] Ji S.-C. *et al.* Experimental determination of the finite-temperature phase diagram of a spin-orbit coupled Bose gas. Nat. Phys. 10, 314–320 (2014) .

[b32] Natu S. S., Li X. & Cole W. S. Striped ferronematic ground states in a spin-orbit-coupled S=1 Bose gas. Phys. Rev. A 91, 023608 (2015) .

[b33] Kibble T. W. B. Topology of cosmic domains and strings. J. Phys. A Math. Gen. 9, 1387 (1976) .

[b34] Zurek W. H. Cosmological experiments in superfluid helium? Nature 317, 505–508 (1985) .

[b35] Lin Y.-J., Perry A. R., Compton R. L., Spielman I. B. & Porto J. V. Rapid production of ^87^Rb Bose-Einstein condensates in a combined magnetic and optical potential. Phys. Rev. A 79, 063631 (2009) .

[b36] Juzeliūnas G. & Spielman I. B. Flux lattices reformulated. New. J. Phys. 14, 123022 (2012) .

[b37] Mewes M.-O. *et al.* Bose-Einstein Condensation in a Tightly Confining dc Magnetic Trap. Phys. Rev. Lett. 77, 416–419 (1996) . 10062807 10.1103/PhysRevLett.77.416

